# Supplementary material for: Interspecies interaction reduces selection for antibiotic resistance in Escherichia coli
Source: Commun Biol. 2023 Mar 27;6:331. doi: 10.1038/s42003-023-04716-2 (PMC10043022; doi:10.1038/s42003-023-04716-2)
Supplement: Supplementary file 2 — Supplementary Information [file 42003_2023_4716_MOESM2_ESM.pdf]

Supplementary information:

Interspecies interaction reduces selection for antibiotic resistance in *Escherichia coli*

Ramith R. Nair[1*] and Dan I. Andersson[1]

[1]Department of Medical Biochemistry and Microbiology, Uppsala University, Uppsala, SE-75123, Sweden

[*]Correspondence to: ramith_nair@hotmail.com

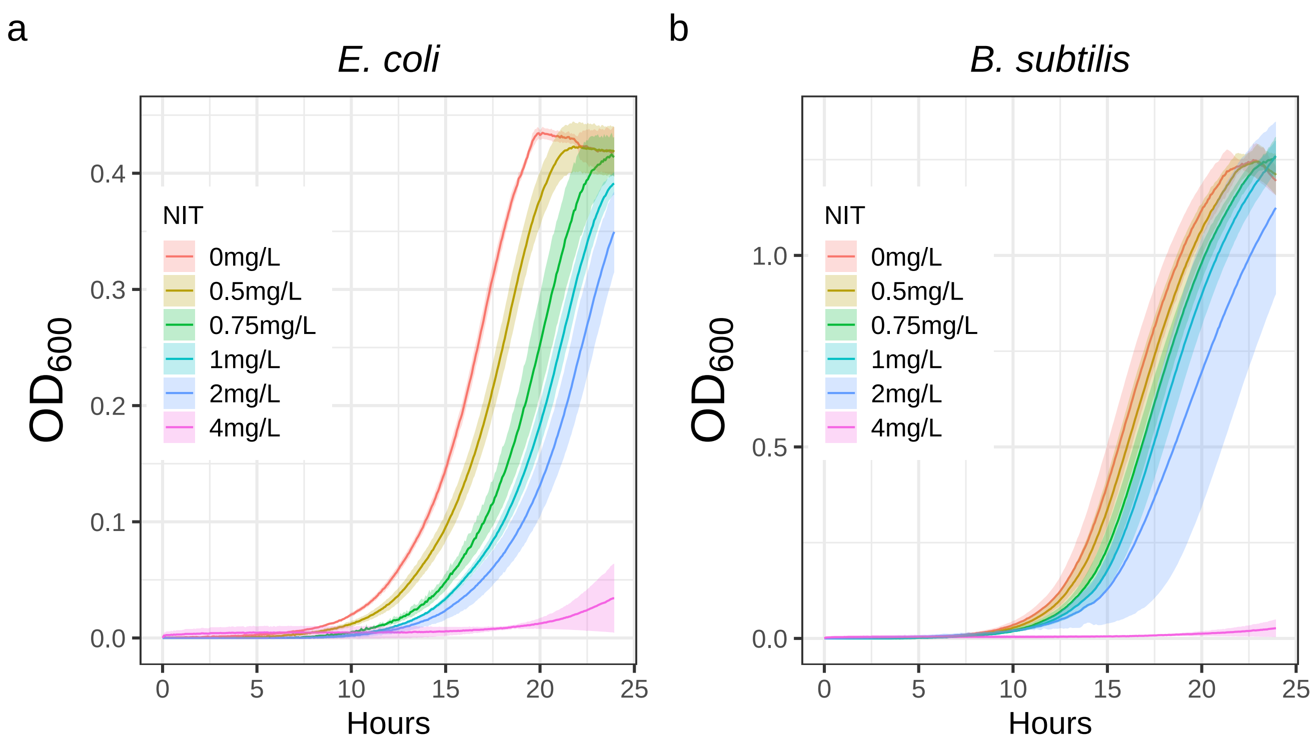

Supplementary Figure 1: **Determination of NIT MIC for *E. coli* and *B. subtilis***. *E. coli* and *B. subtilis* growth depicted as change in OD_{600} at a range of NIT concentrations. Each line is an average from six independent biological replicates. Error bars represent 95% confidence intervals (*t*-distribution, n = 6).

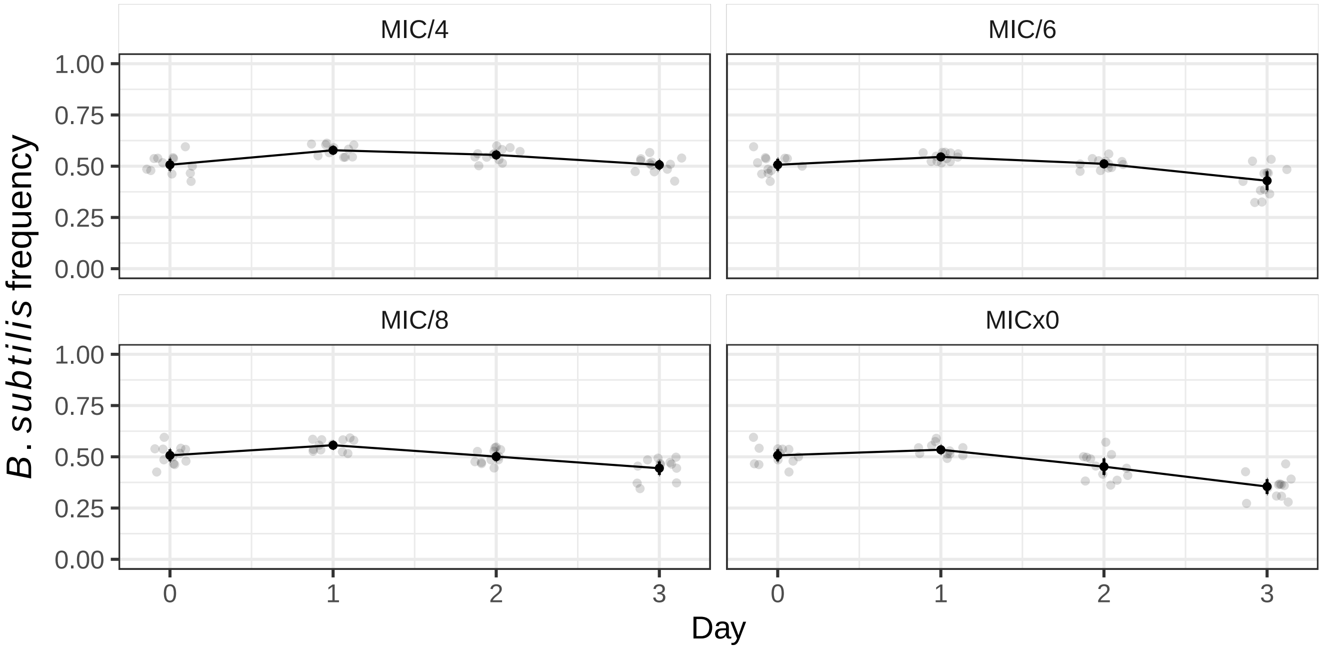

Supplementary Figure 2: **Estimated frequencies of *B. subtilis* in interspecies competitions with *E. coli*.** *B. subtilis* cells are estimated as number of non-fluorescent cells as counted by the flow cytometer. The numbers could be an overestimation due to co-counting of *E. coli* cells that were detected as non-fluorescent and dead cells. Lighter points depict individual replicates, darker points represent means and error bars represent 95% confidence intervals (*t*-distribution, n = 12).

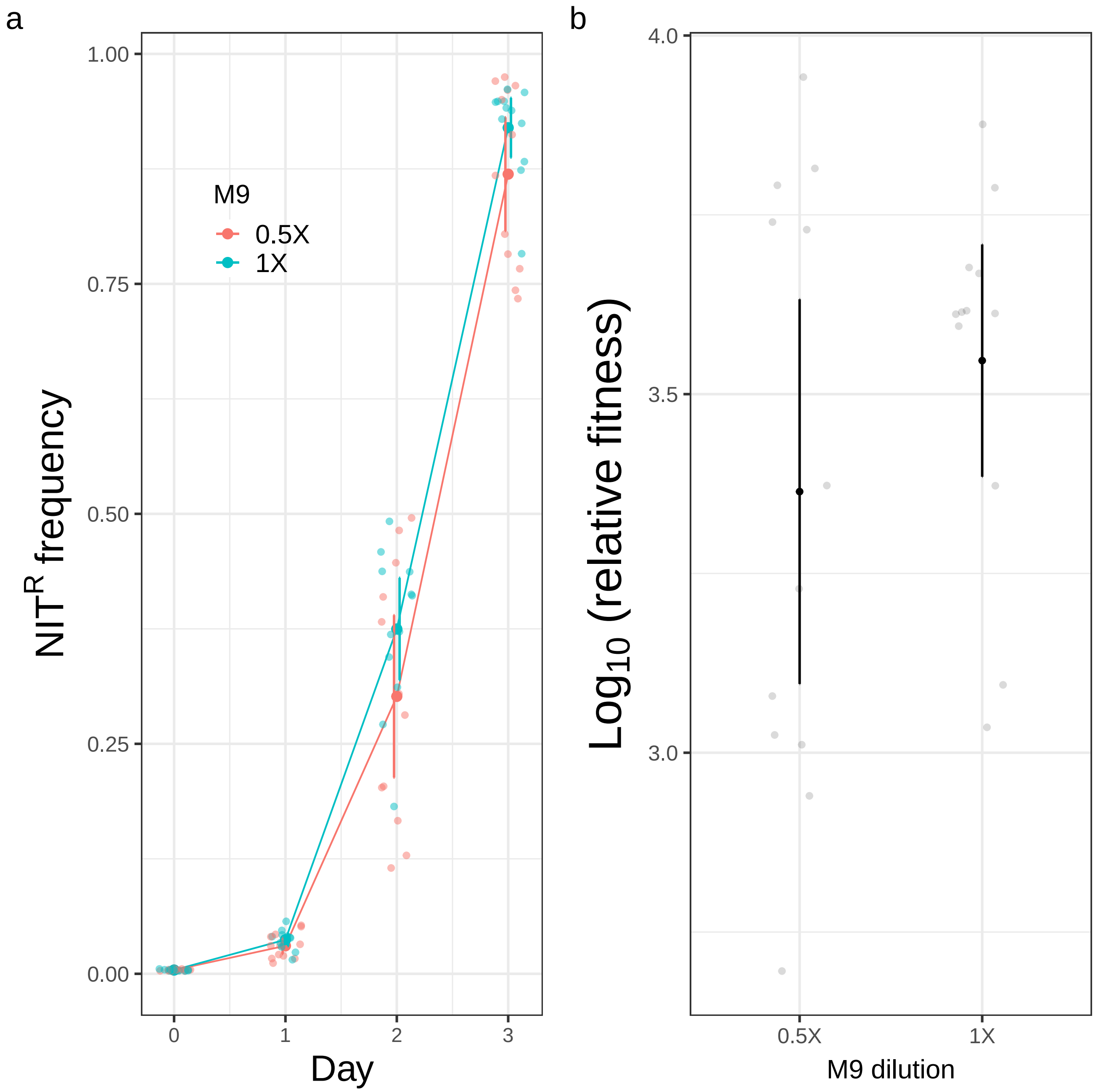

Supplementary Figure 3: **Media dilution does not alter NIT enrichment**. (a) Nitrofurantoin resistance enrichment, and (b) Relative fitness of NIT^R cells in standard and diluted minimal media. Reduction of *E. coli* generations through media dilution has no effect on dynamics or relative fitness of resistant cells. Log-transformed relative fitness value of zero represents no fitness advantage to either NIT^R or NIT^S strains, while positive values indicate an advantage to NIT^R strains. Lighter points depict individual replicates, darker points represent means and error bars represent 95% confidence intervals (t-distribution, n = 12 for both panels).

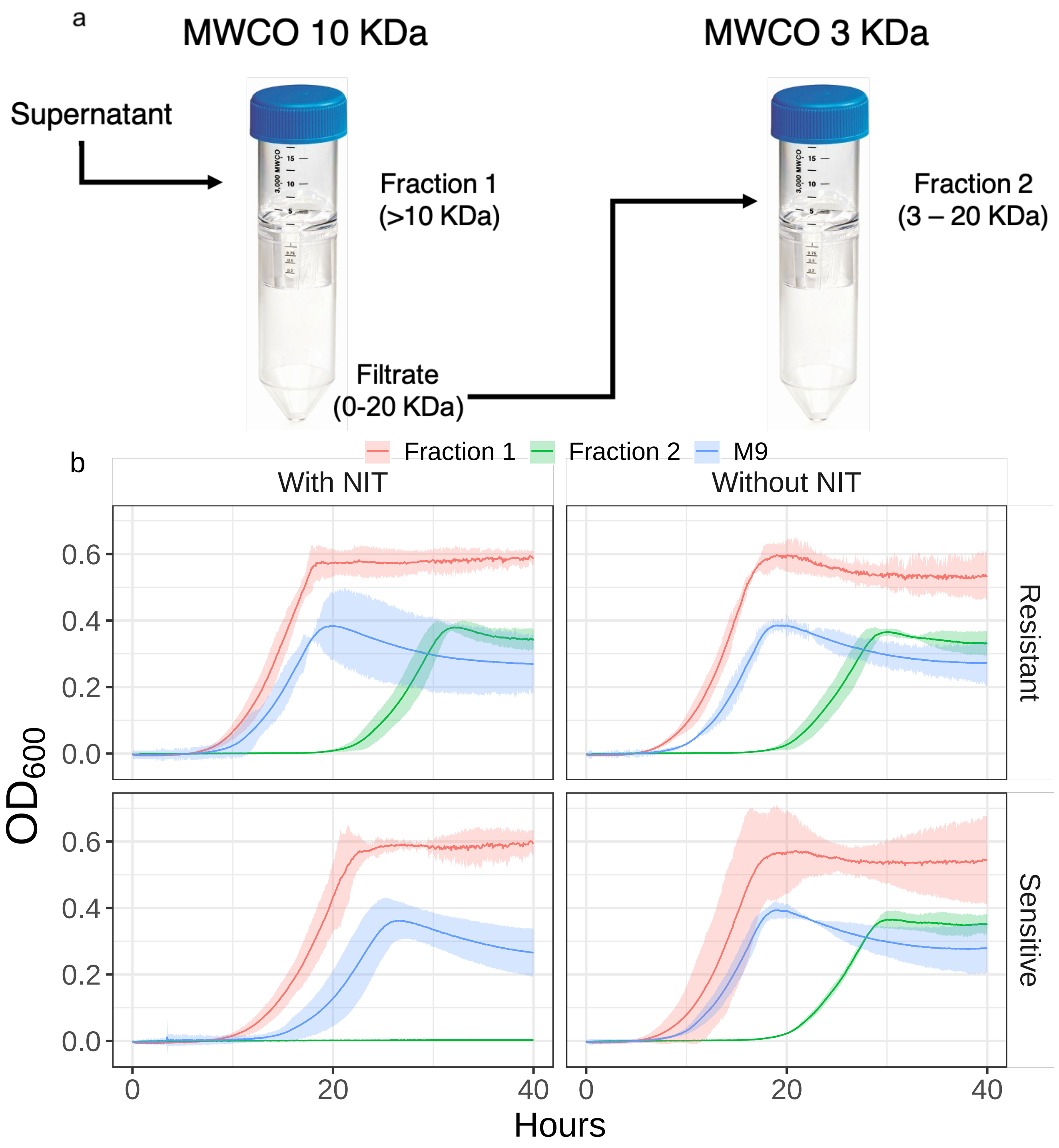

Supplementary Figure 4: **Fractionation and effect of fractions on *E. coli* growth**. (a) Method used to fractionate supernatant in to fraction 1 and fraction 2, (b) Effect of the two supernatant fractions on NIT^S and NIT^R *E. coli* growth in the presence and absence of NIT. Each line is an average from three independent biological replicates. Error bars represent 95% confidence intervals (*t*-distribution, n = 3).

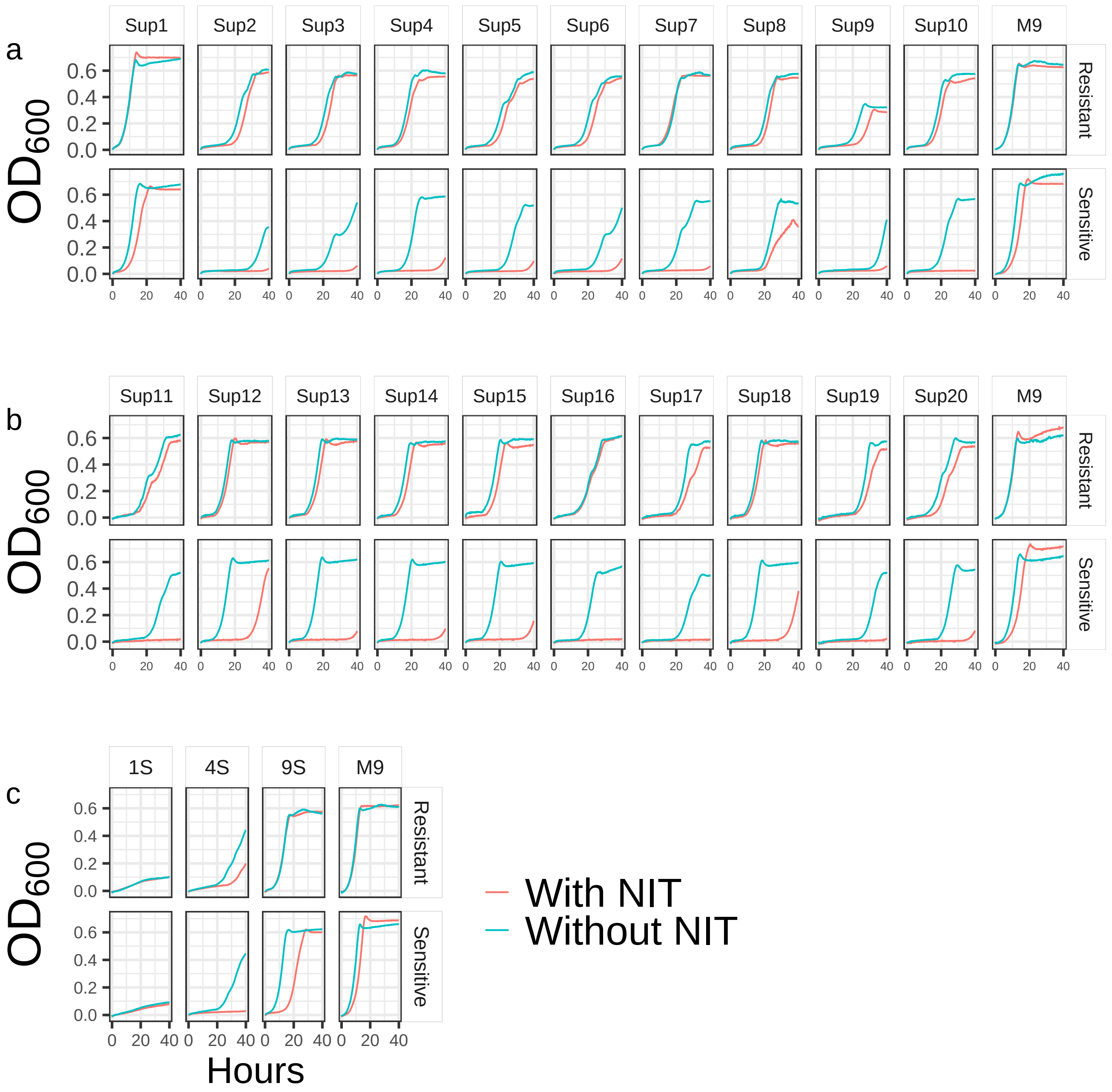

Supplementary Figure 5: **Varied effect of replicate supernatants on *E. coli* growth.** Growth of NIT^S and NIT^R *E. coli* measured as change in optical density in minimal media diluted cell free supernatant from 20 different *B. subtilis* colonies. Measurements for panel (a) and (b) were performed on different days and hence separate media only controls are added for each. For each panel, plots on top row represent those of NIT^R cells and bottom those of NIT^S cells. (C) *E. coli* growth in the presence of the three supernatants selected for proteomic analysis. Each line represents results from one replicate.

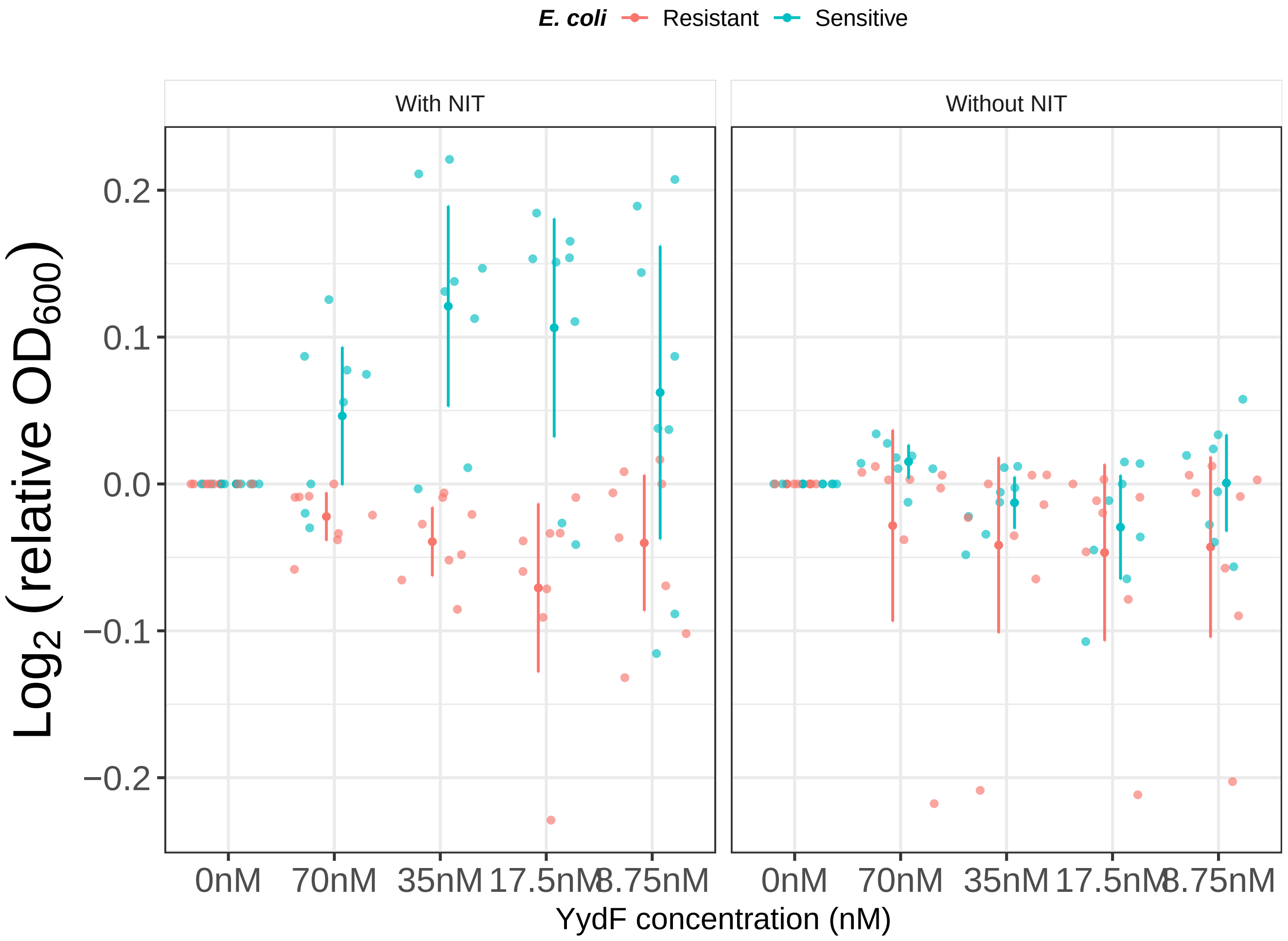

Supplementary Figure 6: **Pre-pro-YydF affects *E. coli* growth.** Effect of different concentrations of peptide on NIT^R and NIT^S *E. coli* growth depicted as log-transformed relative OD_{600} in the presence (left panel) and absence (right panel) of NIT. Error bars represent 95% confidence intervals (*t*-distribution, n = 8).

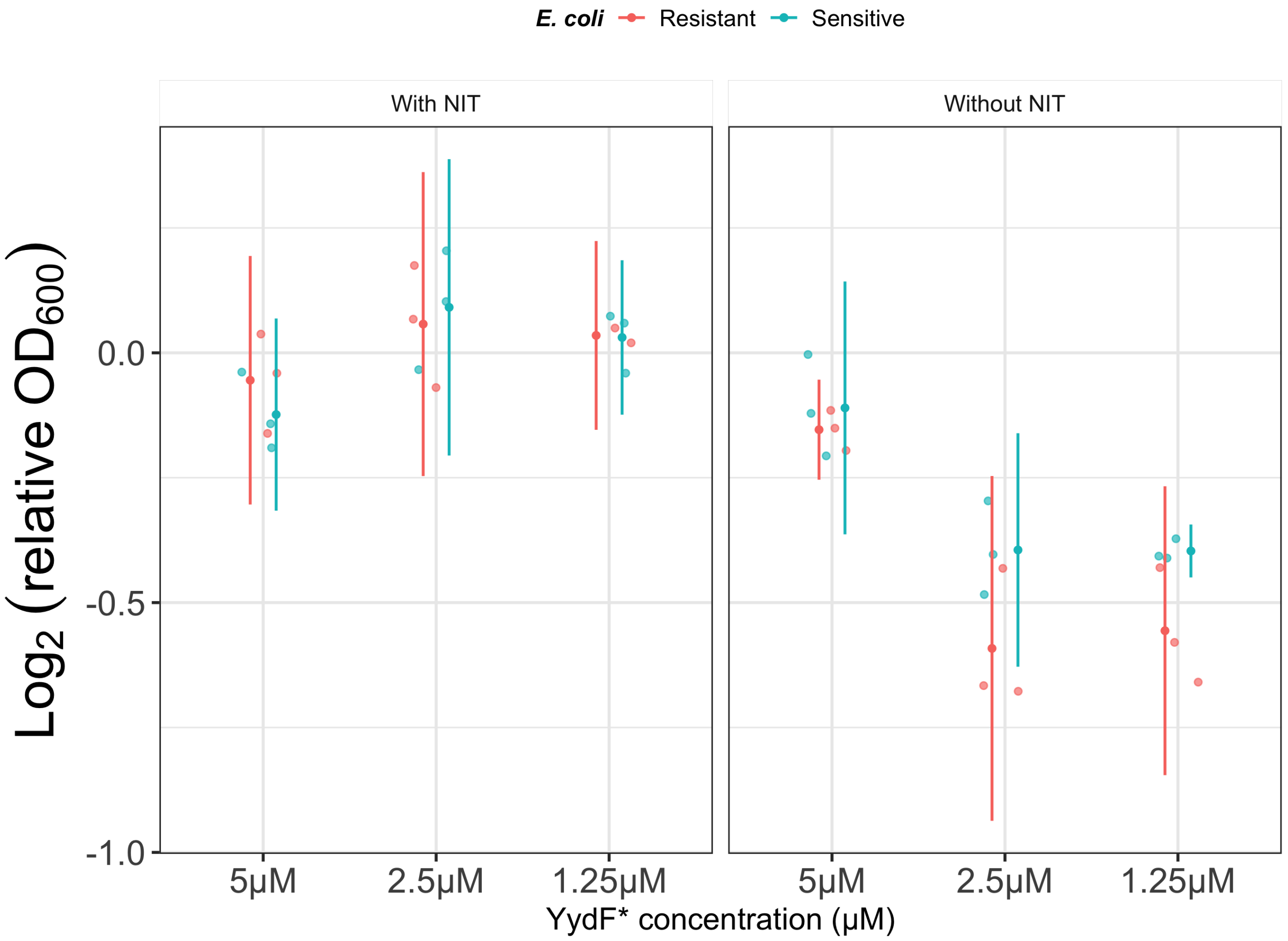

Supplementary Figure 7: **Epipeptide YdyF* does not affect *E. coli* growth**. Effect of different concentrations of epipeptide on NIT^R and NIT^S *E. coli* growth depicted as change in log-transformed relative OD_{600} in the presence (left panel) and absence (right panel) of NIT. Lighter points depict individual replicates, darker points represent means and error bars represent 95% confidence intervals (t-distribution, n = 3).

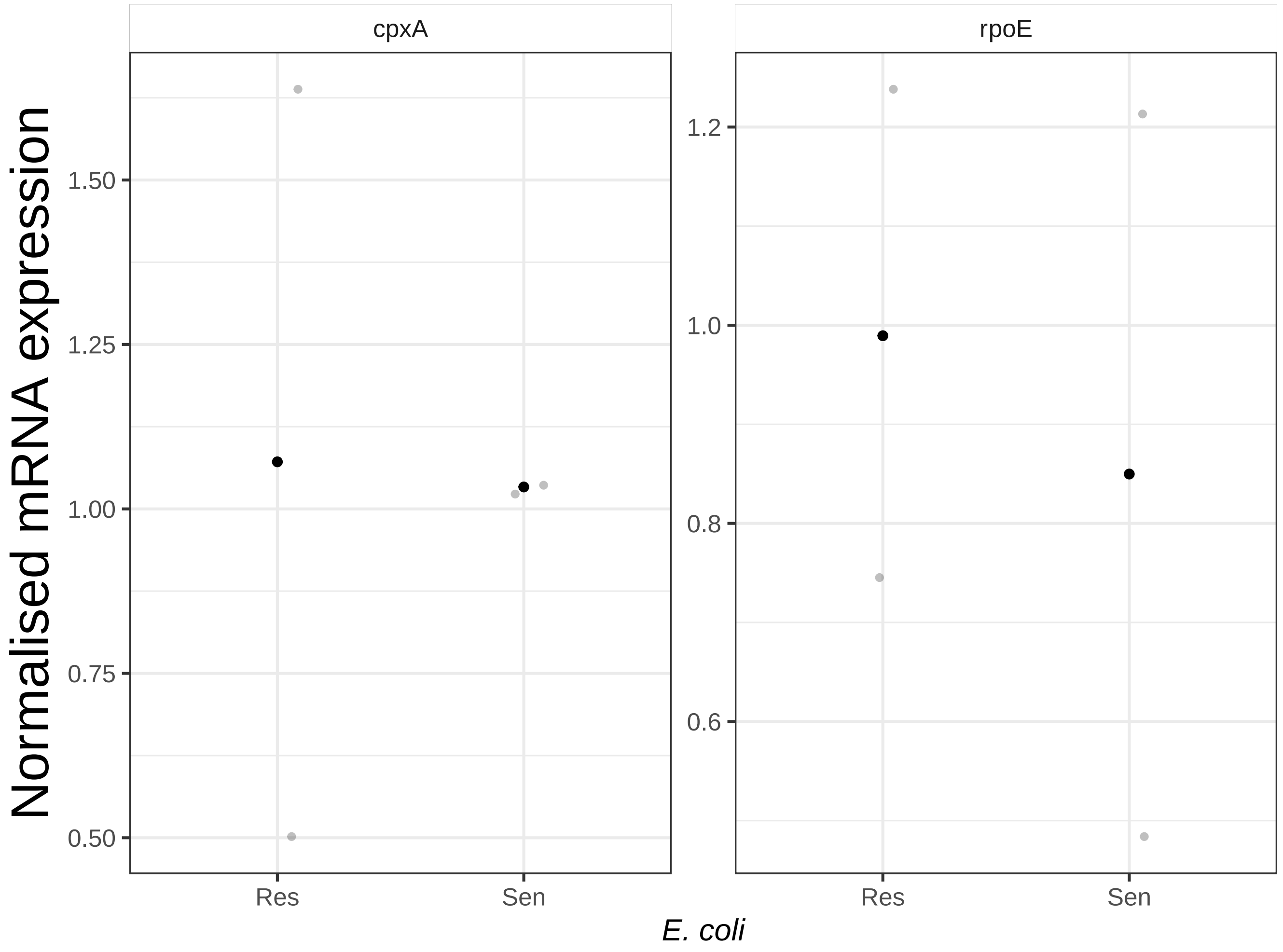

Supplementary Figure 8: **Pre-pro-YydF does not differentially induce envelope stress relates genes in *E. coli***. Differential gene expression of *cpxA* (left panel) and *rpoE* (right panel) measured using qPCR. Expression level for each gene was normalized to house-keeping genes in each case and then ratio of the expression in the presence of the protein over than in the absence were plotted. Darker and lighter points represent means and replicates, respectively (n = 2).

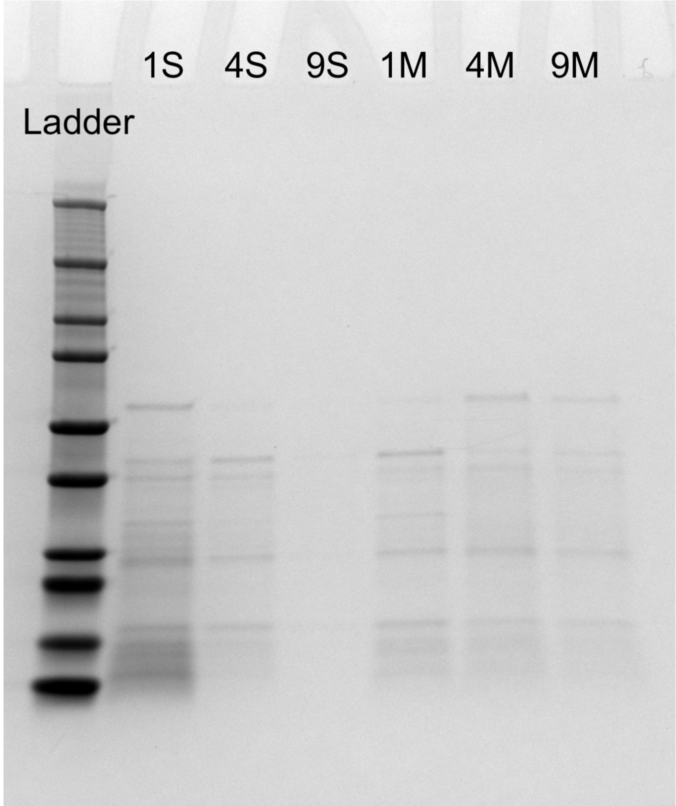

Supplementary Figure 9: **SDS-PAGE of the three *B. subtilis* supernatants analyzed by mass spectrometry**. Protein concentrator column with MWCO of 3 KDa was used to 10X concentrate of proteins from each sample before addition to the well.

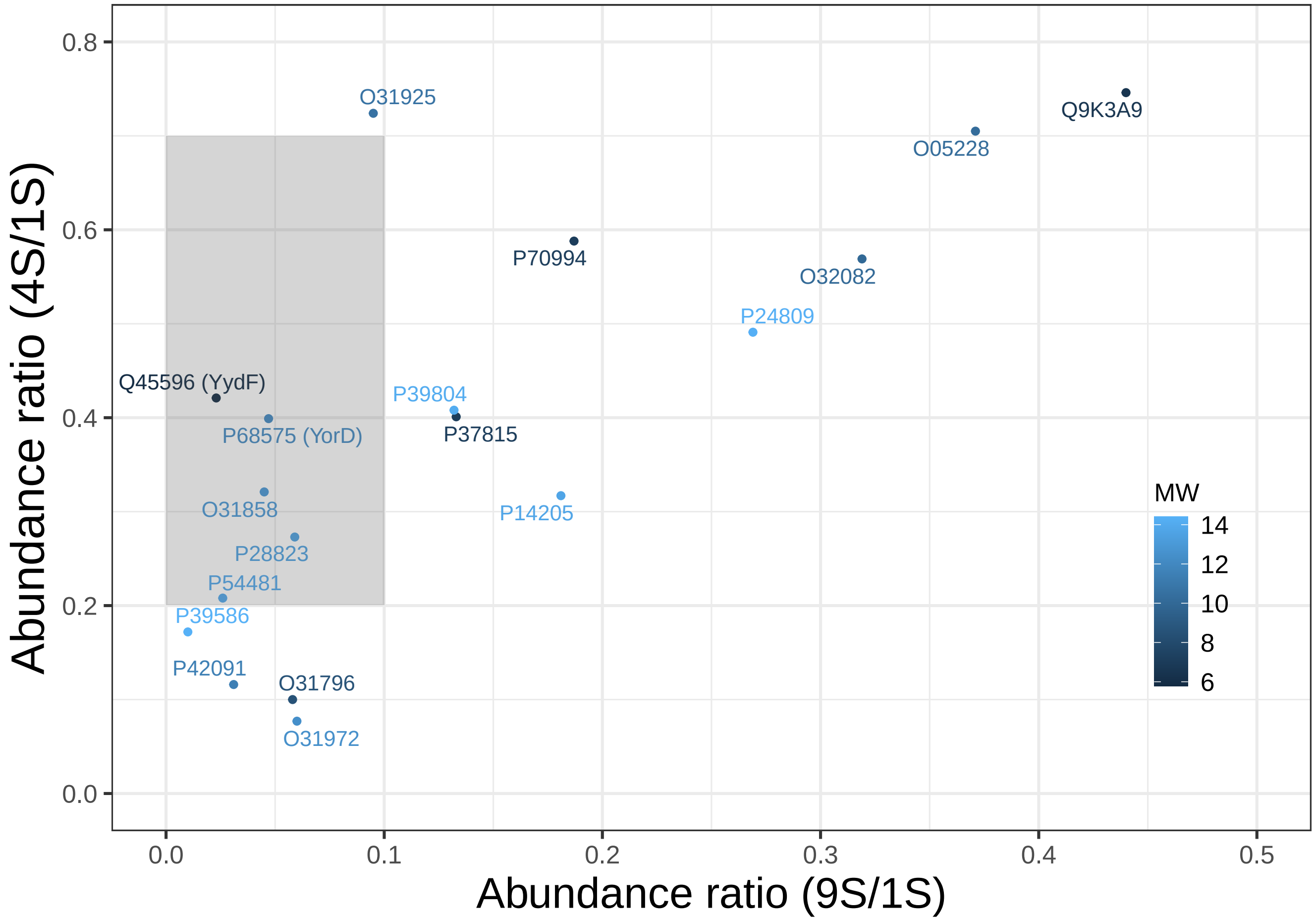

Supplementary Figure 10: **Identification of peptides of interest**. Abundance of proteins in supernatants 9S and 4S relative to that in 1S is plotted on x- and y-axis, respectively. Plot only shows proteins or peptides below 20 KDa. Grey rectangle represents the abundance ratio cut-offs used to identify peptides of interest.
