# Supplementary material for: Interspecies interaction reduces selection for antibiotic resistance in Escherichia coli
Source: Commun Biol. 2023 Mar 27;6:331. doi: 10.1038/s42003-023-04716-2 (PMC10043022; doi:10.1038/s42003-023-04716-2)
Supplement: Supplementary file 3 — Description of Additional Supplementary Files [file 42003_2023_4716_MOESM3_ESM.pdf]

Description of Additional Supplementary Files

File name: Supplementary Data 1
Description: List of all the proteins and peptides identified in the three cell free supernatants from B. subtilis exhibiting differential effects on E. coli growth.
